# Production of Nd and Nd–Fe Alloys from NdCl_3_ by Calciothermic Reduction

**DOI:** 10.3390/ma18050971

**Published:** 2025-02-21

**Authors:** Joo-Won Yu, Yeon-Jun Chung, Jei-Pil Wang

**Affiliations:** 1 Department of Metallurgical Engineering, Pukyong National University, Busan 48513, Republic of Korea; yujw0304@gmail.com; 2 Electrification PE Materials Development Team, Hyundai Motor Co., Hwaseong 18280, Republic of Korea; yjchung@hyundai.com

**Keywords:** metallothermic reduction, neodymium, Nd–Fe alloys, CaCl_2_–KCl–NaCl molten salt, molten metal bath, anhydrous neodymium chloride (III), calcium, carbon-free process

## Abstract

This study presents a metallothermic reduction mechanism for fabricating Nd and Nd–Fe alloys at 850–1050 °C using anhydrous NdCl_3_ and Ca, which have relatively low melting points. Our method decreased the process temperature while improving the recovery rate of Nd using the thermodynamic parameters of the CaCl_2_–KCl–NaCl and Nd–Fe liquid solutions. To reduce the activity of the product (CaCl_2_), the optimal composition of the CaCl_2_–KCl–NaCl molten salt was $X_{{CaCl}_{2}}=0.4\;(X_{KCl}:X_{NaCl}=6:4)$. The molten metal bath (Nd or Nd–Fe) that formed at the bottom of the reaction zone during Nd and Nd–Fe alloy production absorbed metal particles generated in the molten salt during the reaction, thereby facilitating ingot formation. In Nd produced at 1050 °C using 1.2× the stoichiometric amount (by mass) of Ca, the Nd recovery rate was 97.0%. Moreover, in the Nd–Fe alloys produced at 1050 °C targeting eutectic compositions, the Nd recovery rate was 96.3%. Increased Fe contents in the Nd–Fe liquid solution reduced the Nd recovery rates, and the Nd–Fe alloy (Nd recovery rate: 89.8%) was produced at 850 °C, suggesting the possibility of increasing the energy efficiency of the Nd production process. The Nd–Fe alloy produced through this proposed process could be used as a raw material in the NdFeB strip casting process.

## 1. Introduction

Owing to current carbon-neutral goals, the automotive industry has quickly shifted from producing conventional internal combustion engine vehicles to electric vehicle, aiming to reduce greenhouse gas (GHG) emissions [1,2,3]. Consequently, the demands for rare earth metals, such as neodymium–iron–boron (NdFeB) permanent magnets, which are essential materials for electric vehicle motors, and dysprosium and terbium have rapidly increased [4,5]. Among the rare earth metals, Nd is in the highest demand and is primarily produced from neodymium oxide (Nd_2_O_3_) via an electrowinning process commonly used to produce light rare earth elements [6,7,8,9]. This process can be conducted under atmospheric conditions using a moisture-insensitive fluoride-based molten salt (LiF–NdF_3_). However, the use of consumable graphite anodes in the Nd manufacturing process, generates CO/CO_2_ gas and perfluorocarbons such as CF_4_ and C_2_F_6_ [8,9].

As reported in the 2007 IPCC Fourth Assessment Report [10], Table 1 presents the 100-year global warming potential (GWP_100_) of CF_4_ and C_2_F_6_ with values of 7390 and 12,200, respectively. The values correspond to the gases generated during the Nd manufacturing process, which significantly contributes to GWP [11,12].

The GHG emissions from the Nd electrowinning process have been found to vary significantly across previous individual studies [9]. Table 2 presents the emissions of CO, CO_2_, CF_4_, and C_2_F_6_ gases generated during the production of 1 kg of Nd, as well as the carbon dioxide equivalent (CO_2e_) derived from Equation (1) to compare the GWP of each GHG. The emission value of each GHG shown in Table 2 is based on the research presented in Ref. [9], and the CO_2e_ of CO_2_ gas includes the contribution of CO_2_ gas combusted from CO gas emitted from the molten salt during the electrowinning process. Consequently, the total CO_2e_ emissions from the electrowinning process for the production of 1 kg of Nd were determined to be 1.403 kg. To address these environmental concerns, research is underway in the field of electrochemistry to develop inert anode materials to replace graphite anodes [13,14,15,16].

(1) $${CO}_{2e}=\sum_{i}^{N}E_{i}\times {GWP}_{i}$$

where $E_{i}$: emission mass of gas *i* (kg); ${GWP}_{i}$: GWPS of gas *i* relative to CO_2_.

The metallothermic reduction process, which utilizes thermal energy and metals such as Ca and Mg as reducing agents, can be employed to produce Nd from Nd-containing compounds without using carbon. Research on the Nd production process has been conducted in various ways depending on the Nd source, reducing agent, flux, and alloying elements [17,18,19,20]. For example, when Nd_2_O_3_ is used as a raw material in the metal thermochemical reduction process, the number of operation steps is reduced because the only pre-treatment is drying. However, excess flux is required to lower the melting point and activity of the slag (CaO and MgO) generated during the reaction [18,19,20,21]. Therefore, in commercial processes, neodymium fluoride (NdF_3_) obtained via the fluorination of Nd_2_O_3_ has been used as the primary raw material for Nd production. Owing to its low hygroscopicity, NdF_3_ is easy to handle and store, and its Nd recovery rate is ˃97%, rendering it highly applicable in the industry [17]. However, the high melting points of NdF_3_ (1377 °C) and the product CaF_2_ (1418 °C) inhibit the lowering of the process temperature, limiting process energy efficiency.

NdCl_3_ can also be used as an Nd source in the metal thermal reduction process. NdCl_3_ has a lower melting point (758.85 °C) compared to other Nd-containing compounds and reacts with Ca to form Nd and calcium chloride (CaCl_2_), which has a melting point of 772 °C. Thus, NdCl_3_ is a raw material that can improve energy efficiency in terms of process temperature; however, research applying the metallothermic reduction method is limited compared to the reduction method using electrochemistry [22,23,24].

This study aimed to elucidate the thermodynamic mechanism of the calciothermic reduction of NdCl_3_ to yield Nd and identify factors to improve Nd recovery and reduce the process temperature. In the manufacturing of Nd, factors that can enhance its recovery rate from the reaction between NdCl_3_ and Ca, such as the formation of a molten metal bath, CaCl_2_–KCl–NaCl and the amount of Ca used, were examined. Fe, which melts together with Nd metal in the NdFeB strip-casting stage, was selected as an alloying component of Nd to reduce the process temperature [25]. To fabricate the Nd–Fe alloy, the effects of Fe composition in the Nd produced from the reaction of NdCl_3_ and Ca and the reduction of process temperature on the Nd recovery rate were analyzed.

NdCl_3_ is typically synthesized by reacting Nd_2_O_3_ with hydrochloric acid (HCl) to produce the NdCl_3_·6H_2_O hydrate during the crystallization process [26]. Subsequently, NdCl_3_·6H_2_O is converted to NdOCl at temperatures ˃371 °C [27]. In the metallothermic reduction process, the formation of NdOCl can induce the formation of high-melting point slag and oxygen contamination in Nd. Moreover, it can increase the process temperature due to its higher melting point than NdCl_3_. Considering these issues, NdCl_3_ produced from an aqueous solution may not be suitable for use in the metallothermic reduction process.

Therefore, anhydrous NdCl_3_ produced via the chlorination reaction between Nd_2_O_3_ and ammonium chloride (NH_4_Cl), which was conducted in a previous study, was used in this study [27]. Generally, NH_4_Cl is prepared through the acid-base reaction between ammonia (NH_3_) gas and hydrogen chloride (HCl) gas [28], while NH_3_ gas is synthesized from nitrogen (N_2_) and hydrogen (H_2_) gas through the Haber–Bosch process [29]. Additionally, H_2_ gas is typically produced using methane (CH_4_), which is the primary component of natural gas, and CO_2_ is emitted during this process [30]. Thus, using NH_4_Cl for the production of NdCl_3_ can yield emissions of CO/CO_2_ gas.

In this study, the GHG emissions from the manufacturing processes of raw materials required to produce 1 kg of Nd were investigated and compared with the existing Nd electrowinning process. Additionally, the possible regeneration of NH_4_Cl by capturing NH_3_ gas generated during the chlorination process was examined.

## 2. Materials and Methods

### 2.1. Materials

**Figure 1:**
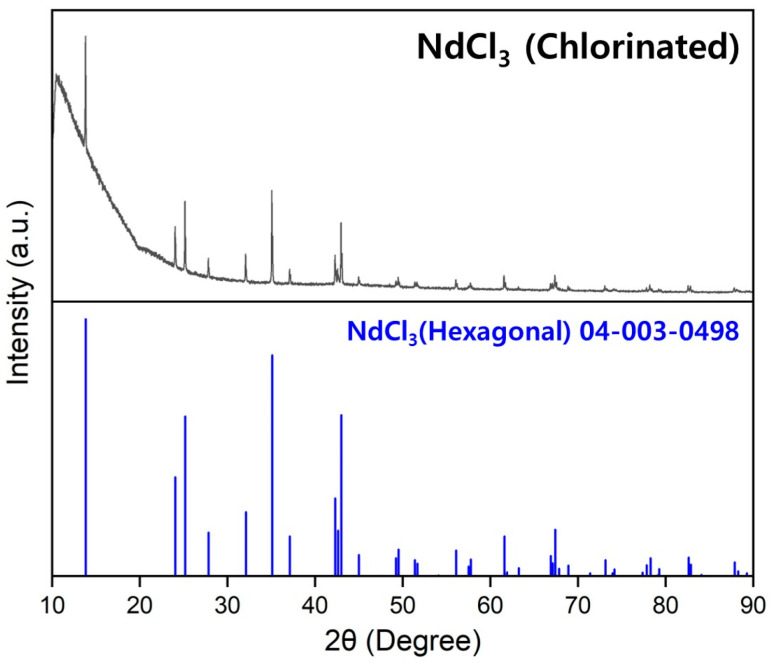
XRD pattern of NdCl_3_ prepared via the chlorination reaction of Nd_2_O_3_ and NH_4_Cl [27].

Anhydrous NdCl_3_ used in the production of Nd and the Nd–Fe alloys was prepared via the chlorination reaction of Nd_2_O_3_ (Alfa Aesar, Haverhill, MA, USA, 99%) and NH_4_Cl (JUNSEI, Tokyo, Japan, 98.5%) [27]. Figure 1 shows the X-ray diffraction (XRD) pattern of the synthesized anhydrous NdCl_3_ (purity: 98.65%). To increase its purity, it was melted at 780 °C for 20 min to produce a bulk form and then ground to obtain a powder with a particle size of ˂100 μm. Ca (Daejung, Siheung, Republic of Korea, 97.6%) was used as a reductant for NdCl_3_, and Fe (Duksan, Ansan, Republic of Korea, 99.4%), KCl (Duksan, 99%), and NaCl (Duksan, 99.5%) were used to form the Nd–Fe and CaCl_2_–KCl–NaCl solutions. To remove moisture, KCl and NaCl were dried under vacuum (<5 × 10^−4^ Torr) at 150 °C for 12 h before use. A molten metal bath was formed at the lower part of the reaction zone using Nd metal (KSM, Cheongju, Republic of Korea, 98.8%) and Fe powder (Duksan, 99.4%) as absorption sources for the Nd and Nd–Fe produced during the reaction. The Nd metal was remelted using vacuum arc melting to prevent oxidation during the cutting process and to enhance purity.

### 2.2. Experimental Apparatus

**Figure 2:**
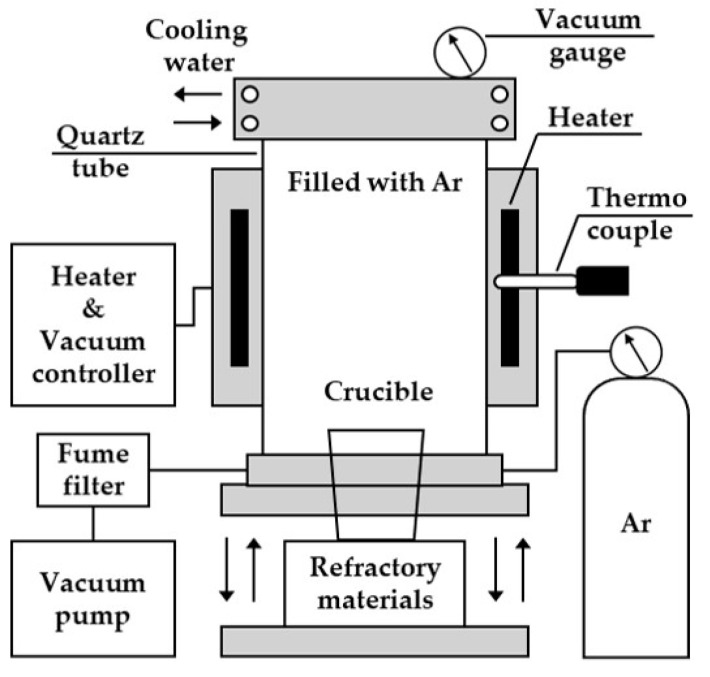
Schematic diagram of the vacuum drying furnace used to remove moisture from the reagents.

Figure 2 shows a schematic diagram of the vacuum drying furnace used to remove moisture from the raw materials during the metallothermic reduction process. The reaction chamber comprised a quartz tube surrounded by a heater, with a thermocouple attached to the side of the heater. The upper part of the chamber that was connected to the quartz tube was cooled by circulating water to prevent heat-induced damage. The crucible was placed inside the reaction chamber through the movement of an up-and-down plate mechanism connected to the bottom of the chamber. Refractory material was installed at the bottom of the crucible, considering the position of the heating zone. A vacuum was created inside the chamber using a vacuum pump connected to the bottom of the reaction chamber. Following reaction completion, Ar gas was injected into the chamber using a vacuum regulator to restore atmospheric pressure.

**Figure 3:**
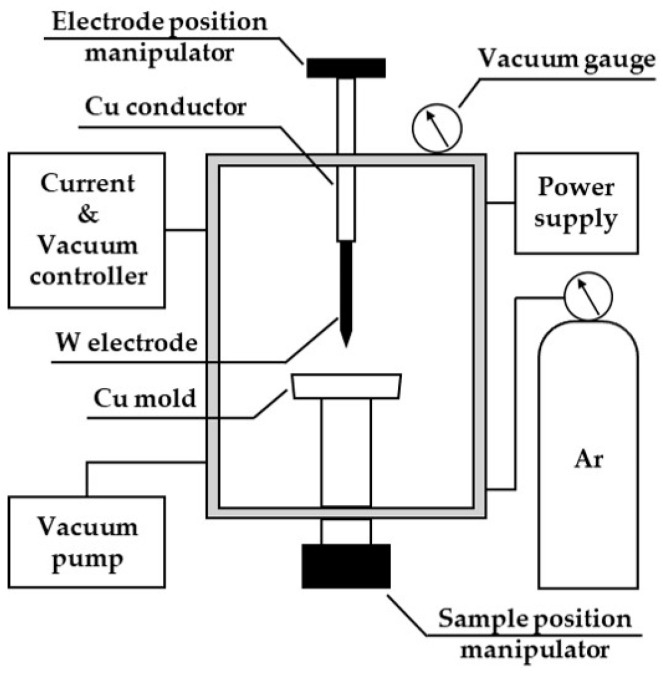
Schematic diagram of the vacuum arc melting furnace used for the remelting of Nd metal to form a molten metal bath.

Figure 3 presents a schematic diagram of the vacuum arc melting furnace used to produce Nd, which was utilized as a molten metal bath. A water-cooled Cu mold was installed at the center of the chamber for melting the sample, and a device for adjusting the position of the sample was connected to its lower part. An electrode position adjuster was mounted above the Cu conductor, and a non-consumable W electrode was connected at the bottom. To create a vacuum–Ar atmosphere, the chamber was equipped with a vacuum pump, vacuum gauge, and Ar cylinder. The current and vacuum level were controlled using a controller mounted on the side of the chamber.

**Figure 4:**
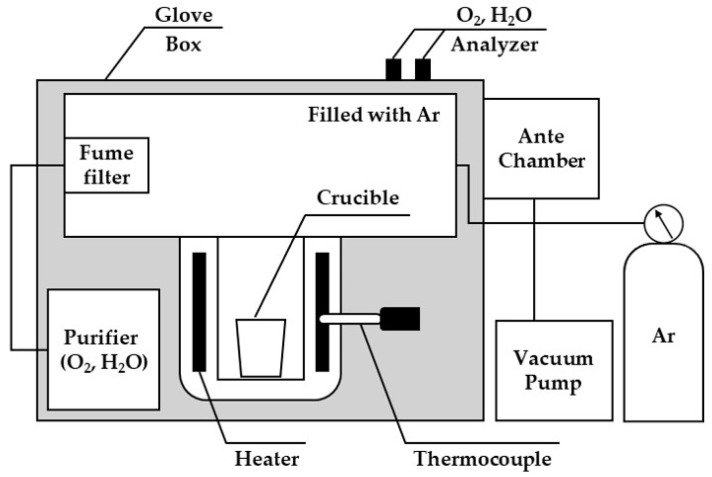
Schematic diagram of the glove box used in the metallothermic reduction under an Ar atmosphere [27].

Figure 4 shows a schematic diagram of the experimental setup in a glove box filled with Ar gas. The primary compartment of the glove box was purged using Ar gas, and O_2(g)_ and H_2_O_(g)_ concentrations were controlled at <1 ppm using the purifier. The heater was attached to the reactor compartment located at the bottom of the primary compartment of the glove box, and a thermocouple was installed near the reactor crucible. The raw materials were maintained in an antechamber under vacuum (1 × 10^−5^ bar) for 6 h to remove air present in the powder and then charged into the reactor.

**Figure 5:**
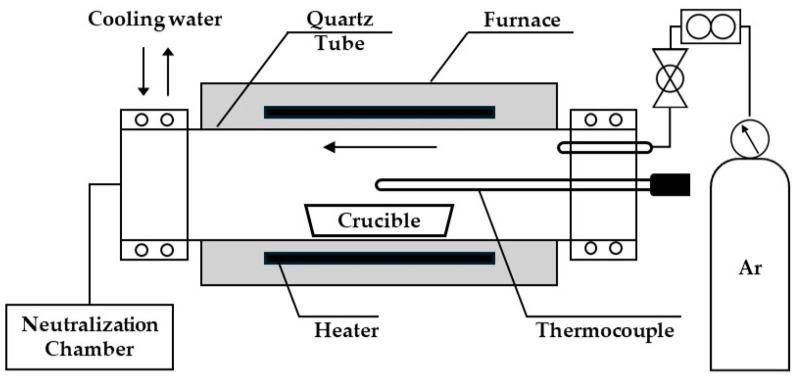
Schematic diagram of the horizontal furnace used to capture NH_3_ gas generated during the chlorination reaction of Nd_2_O_3_ and NH_4_Cl [27].

Figure 5 shows a schematic diagram of the horizontal furnace used to capture NH_3_ gas generated during the chlorination process with Nd_2_O_3_ and NH_4_Cl. The reaction tube consisted of a quartz tube (L600 mm × D49 mm × T2 mm) and a water-cooled SUS covered with an O ring at both ends. A quartz tube was used to prevent corrosion caused by salt in the chlorination reaction area. The Ar gas (>99.99%) line consisted of a flow meter (max: 500 mL/min) to regulate the gas supply and a check valve to prevent corrosion. An R-type thermocouple coated with alumina was used to prevent salt corrosion. The area connected to the metal cover was sealed with Teflon tape to prevent leakage and then coupled with an O-ring. The gas outlet line was connected to a neutralization chamber containing HCl to capture the NH_3_ generated during the reaction.

### 2.3. Experimental Analysis

**Figure 6:**
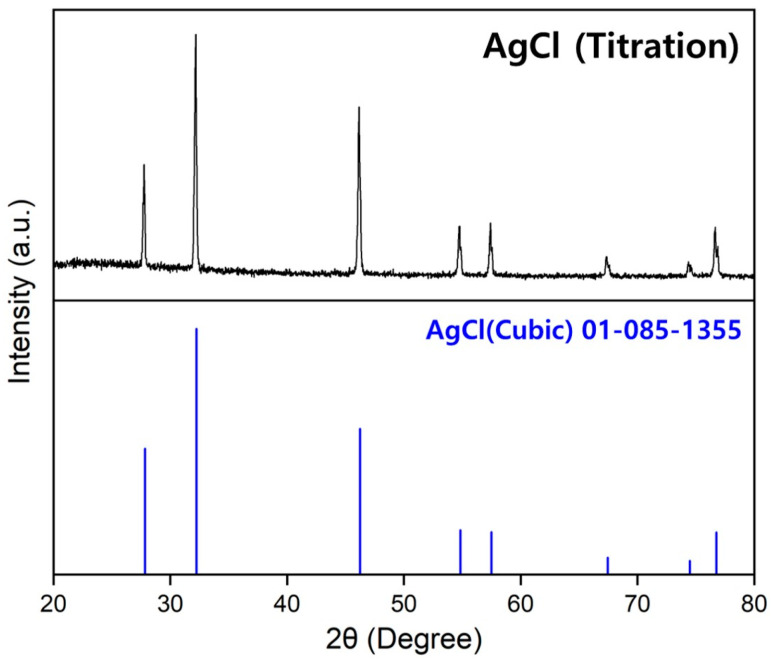
XRD pattern of the precipitate produced during the AgNO_3_ titration process of Nd and the Nd–Fe alloys manufactured via the metallothermic reduction process.

The concentrations of Ca, K, Na, and W present in Nd and the Nd–Fe alloys were analyzed using inductively coupled plasma optical emission spectroscopy (Agilent 5800, Santa Clara, CA, USA), and the concentration of Cl was determined using AgNO_3_ titration. Figure 6 shows the XRD pattern of the precipitate generated following titration, with monophase AgCl detected. The C and O concentrations in the raw materials (Nd, Fe, and Ca) and the products (Nd and Nd–Fe alloys) of the metallothermic reduction process were analyzed using a carbon/sulfur analyzer (Eltra, CS-2000, Retsch-Allee, Haan, Germany) and oxygen/nitrogen analyzer (ON, Eltra, ELEMENTRAC ON-p2, Retsch-Allee, Haan, Germany), respectively. Scanning electron microscopy (SEM) images of the Nd–Fe alloy were obtained using a Schottky microscope (JEOL, JSM-IT800SHL, Tokyo, Japan). The phase composition of the chlorides (CaCl_2_ and CaCl_2_–KCl–NaCl) generated in the metallothermic reduction process was measured using an X-ray diffractometer (Bruker D8 Advance A25 Plus, Billerica, MA, USA) with Cu Kα1 radiation (λ = 0.15406 nm) and an airtight holder (Bruker model). A 2θ range of 0–90° was scanned at a rate of 3°/min. The phase composition of the substances formed through the acid-base reaction between NH_3_ gas, which was generated during the chlorination process, and HCl was analyzed using a Rigaku Ultima IV powder X-ray diffractometer (Tokyo, Japan) with Cu Kα_1_ radiation (λ = 0.154060 nm) over a 2θ range of 0–90° at a scan rate of 3°/min.

### 2.4. Experimental Procedure

#### 2.4.1. Drying of the Raw Materials

The quartz crucible containing the KCl and NaCl powders was placed inside the chamber using a vertically movable plate mounted at the lower section of the vacuum drying furnace. A vacuum regulator was used to create a negative pressure inside the chamber (ultimate vacuum: 5 × 10^−4^ Torr), and the temperature was increased to 150 °C at a heating rate of 5 °C/min. After drying for 12 h, Ar gas was injected into the chamber using the vacuum regulator. To minimize contact between the dried powders and moisture or oxygen in the air, they were immediately transferred to the antechamber of a glove box. Following drying, the powders were ground using a mortar to remove weakly bonded agglomerates formed during the process.

#### 2.4.2. Vacuum Arc Melting

Considering the initial volume of the Nd metal, it was crushed using a hydraulic cutter, and the oxide layer on the metal surface was removed by polishing. After the processed Nd sample was placed in a Cu mold, a negative pressure (ultimate vacuum: 5 × 10^−5^ Torr) was formed inside the chamber for 2 h using the vacuum controller. The pressure inside the chamber was set to the optimal pressure for arc formation (vacuum pressure: −0.05 MPa) via the injection of Ar gas. After setting the voltage (23 V) and current (230 A) to form the arc, the melting of the sample proceeded by controlling the sample and electrode position controllers. Ar gas was injected to bring the chamber pressure to atmospheric pressure, the sample was recovered, and the Nd metal was produced (80–100 g).

#### 2.4.3. Metallothermic Reduction

**Figure 7:**
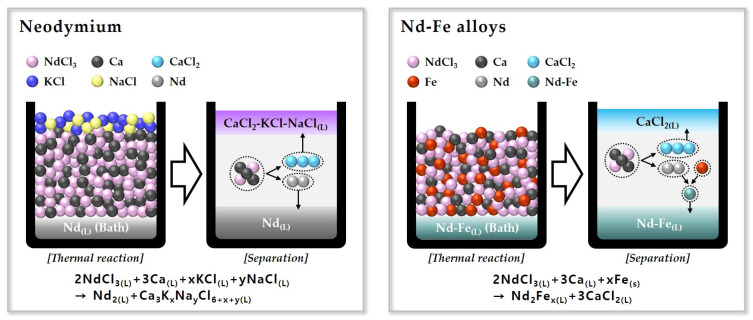
Schematic diagram of the metallothermic reduction process to produce Nd and the Nd–Fe alloys.

Figure 7 shows a schematic diagram of the metallothermic reduction process to produce Nd and Nd–Fe alloys.

- Manufacture of Nd: Approximately 100 g of arc-melted Nd was added to the bottom of a W crucible. After weighing the raw materials, mixed powders comprising NdCl_3_ and Ca and KCl and NaCl were sequentially added (Table 1). To minimize reactant evaporation during the reaction process, the upper part of the crucible was sealed with a W cover. The reactor was heated to 1050 °C at a rate of 5 °C/min and maintained for 4 h, with stirring performed at one-hour intervals. Following reaction completion, the liquid product in the crucible was poured into a mold. After natural air cooling, the metal and salt were separated using tools and a mortar, and metal particles of ˃45 μm were recovered following salt crushing. During analytical pre-treatment, cutting processing was conducted inside the glove box to avoid metal oxidation. The CaCl_2_ and CaCl_2_–KCl–NaCl crushed to ˂45 μm were sealed in an airtight holder during XRD analysis, considering the reaction with moisture. The W crucible and stirring rod were cleaned with HCl (35%) and reused in subsequent experiments;
- Manufacture of the Nd–Fe alloys: Before adding the reactants, homogenization of the Nd–Fe molten metal bath was performed. After adding Nd and Fe to the W crucible as shown in Table 1, it was heated above the melting point of Nd (to 1100 °C) at a heating rate of 5 °C/min and maintained for 20 min. Stirring was conducted before extracting the crucible, which was naturally air cooled inside a glove box. After adding the measured NdCl_3_, Ca, and Fe mixed powder to the top of the Nd–Fe alloy layer (Table 1), the temperature was increased to proceed with the reaction. Subsequent steps were performed in the same manner as the Nd manufacturing process.

#### 2.4.4. Capture of NH_3_ Gas

Briefly, 5 and 7.4796 g of dried Nd_2_O_3_ and NH_4_Cl powders were mixed and placed in a quartz crucible, which was positioned at the center of a reaction tube. The Ar gas flow rate was maintained at 500 mL/min for 30 min to remove gaseous impurities from the reaction tube. Subsequently, the temperature was increased to 400 °C at a heating rate of 5 °C/min under Ar flow. This condition was maintained for 2 h, and the furnace was naturally cooled to room temperature. Finally, the solution containing the gas that was collected in the neutralization chamber was dried at 150 °C for 24 h.

### 2.5. Variables of the Metallothermic Reduction Process

- Production of Nd: Considering the Gibbs free energy of the CaCl_2_–KCl–NaCl liquid solution derived from the thermodynamic analysis and the melting point of Nd, the reaction temperature was set at 1050 °C. The amounts of reactants were determined based on 100 g of NdCl_3_. The reductant Ca was used up to a maximum of 31.1859 g (×1.3), and the mass ratio (×1.n) was increased based on the stoichiometric ratio (23.9891 g) with NdCl_3_. The composition of KCl and NaCl in the CaCl_2_–KCl–NaCl molten salt was derived from the thermodynamic analysis using FactSage 8.3. The Nd metal used in the molten metal bath was close to 100 g;
- Production of the Nd–Fe Alloys: Considering the Gibbs free energy of the Nd–Fe liquid solution derived from the thermodynamic analysis and the melting point of the Nd–Fe alloy, the reaction temperature was set at 850–1050 °C. The composition of Nd (57.5584 g), which was theoretically producible from 100 g of NdCl_3_ and Fe, corresponded to the composition of Nd and Fe in the molten metal bath. Thus, the theoretical compositions of the Nd–Fe solution formed from the reactants, molten metal bath, and final product, the Nd–Fe alloy, were identical;
- Controlled Variables: The reaction time was fixed at 4 h under all conditions, and stirring was performed at one-hour intervals during the reaction. To minimize the effects of moisture and oxygen, an Ar atmosphere (O_2_: 1 ppm, H_2_O: 1 ppm) was maintained. The experimental conditions and equilibrium compositions are shown in Table 3 and Table 4, respectively.

## 3. Results and Discussion

### 3.1. Thermodynamic Considerations

#### 3.1.1. Standard Gibbs Free Energy

Table 5 shows the temperature-dependent changes in the standard Gibbs free energy for the stoichiometric reaction between NdCl_3_ and Ca. The temperature range was investigated starting from the melting point of NdCl_3_ at 758 °C (1032 K) to consider the solid–liquid reaction between the reactants. The upper temperature limits for each range corresponded to the phase transition temperatures of the components. According to the Gibbs free energy results, the products were more stable than the reactants in all the investigated temperature ranges. For example, at temperatures of 800 and 1100 °C, the changes in standard Gibbs free energy were −341.76 and −337.13 kJ, respectively.

#### 3.1.2. CaCl_2_–KCl–NaCl Molten Salt System

KCl and NaCl were selected as components of the liquid solution to reduce the activity of CaCl_2_. NaCl started reacting with Ca at temperatures >700 °C under standard conditions. However, at 800–1100 °C, NaCl had a lower reaction driving force (${∆G}_{1073K}^{o}=-6.9\;kJ,{∆G}_{1373K}^{o}=-38.7\;kJ)$ with Ca compared to NdCl_3_. To determine the composition of the CaCl_2_–KCl–NaCl molten salt, its thermodynamic properties were examined, and the analysis was performed using the FTsalt database in FactSage 8.3. The relative integral molar Gibbs free energy of mixing and excess Gibbs free energy of the CaCl_2_–KCl–NaCl liquid solution were calculated according to Equations (2) and (3), respectively.

(2) $${∆G}^{M}=\sum_{i=1}^{C}x_{i}{∆\bar{G}}_{i}^{M}=RT\sum_{i=1}^{C}x_{i}lna_{i}$$

where $x_{i}$: mole fraction of component *i*; R: gas constant (J/mol·K); T: absolute temperature (K); and $a_{i}$: activity.

(3) $$G^{XS}=\sum_{i=1}^{C}x_{i}{\bar{G}}_{i}^{XS}=RT\sum_{i=1}^{C}x_{i}ln\gamma_{i}$$

where $\gamma_{i}$: activity coefficient of component *i*.

**Figure 8:**
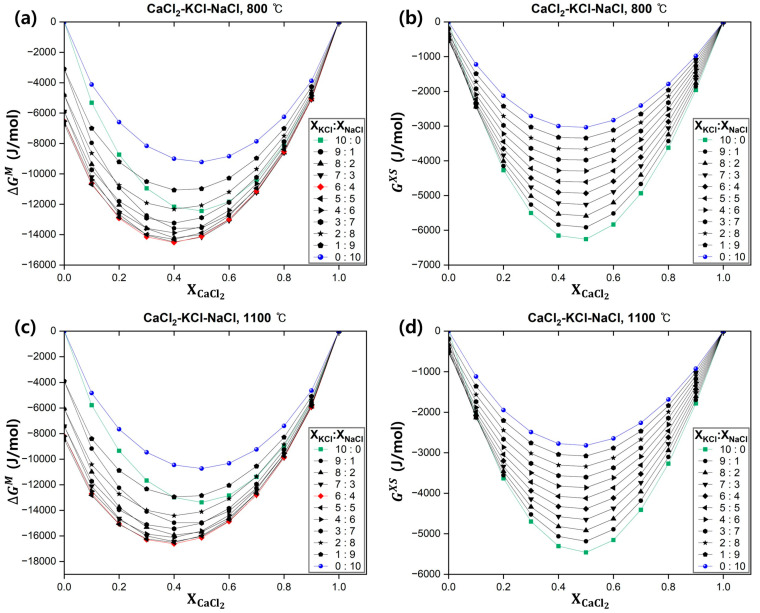
Gibbs free energy values of the CaCl_2_–KCl–NaCl liquid solution with changes in system temperature and composition: (**a**) Relative integral molar Gibbs free energy of mixing at 800 °C; (**b**) Excess Gibbs free energy at 800 °C; (**c**) Relative integral molar Gibbs free energy of mixing at 1100 °C; (**d**) Excess Gibbs free energy at 1100 °C.

The Gibbs free energy values were investigated at 800–1100 °C, considering the melting points of the reactants and products. The Gibbs free energy values of the solution at the lower and upper temperature limits are presented in Figure 8. Notably, the Gibbs free energy of the solution exhibited the same behavior between 800 and 1100 °C. Figure 8 shows the Gibbs free energy of the CaCl_2_–KCl–NaCl liquid solution as a function of the molar fraction of CaCl_2_. The curves were distinguished by the various cross sections of KCl and NaCl. According to Figure 8a,c, the Gibbs free energy of the CaCl_2_–KCl–NaCl liquid solution in the temperature range of 800–1100 °C was more stable than that of the CaCl_2_–KCl and CaCl_2_–NaCl liquid solutions and decreased as the system temperature increased. Thus, as the ternary system formed, the relative integral molar entropy of mixing increased. Consequently, the Gibbs free energy was more affected by the entropy–temperature term. According to Figure 8b,d, the CaCl_2_–KCl–NaCl liquid solution was more stable than the ideal solution across all temperature and composition ranges examined. Additionally, the excess Gibbs free energy increased with increasing system temperature. This result was attributed to a decrease in the non-ideal behavior of the molten salt system due to the disordering effect caused by the increased temperature. Thus, the composition of the CaCl_2_–KCl–NaCl liquid solution with the minimum Gibbs free energy value at 800–1100 °C was $X_{{CaCl}_{2}}=0.4\;(X_{KCl}:X_{NaCl}=6:4)$, and the corresponding thermodynamic parameters are presented in Table 6.

#### 3.1.3. Nd–Fe Binary System

**Figure 9:**
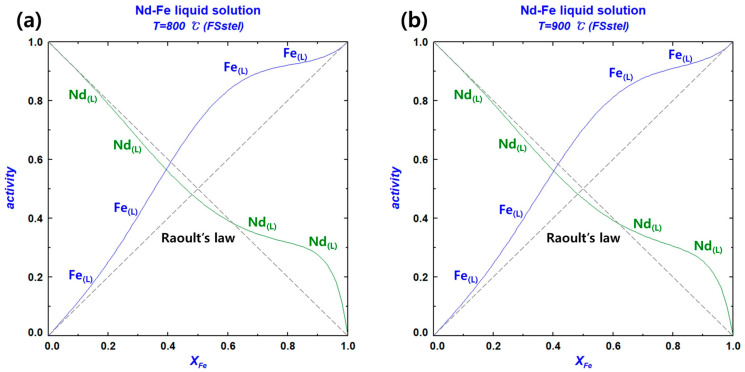

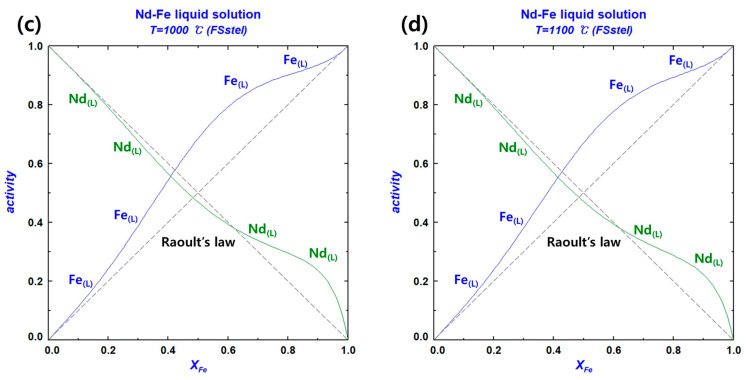
Activity–composition diagrams of Nd and Fe in the Nd–Fe liquid solution at different temperatures: (**a**) 800 °C; (**b**) 900 °C; (**c**) 1000 °C; and (**d**) 1100 °C.

The thermodynamic analysis of the Nd–Fe liquid solution was performed using the FSstel database in FactSage 8.3. The activities of Nd and Fe in the Nd–Fe liquid solution were calculated within the temperature range of 800–1100 °C. The activities of the two components as a function of temperature and composition are shown in Table 7. The activity–composition diagrams of Nd and Fe as a function of $X_{Fe}$ are presented in Figure 9. According to the calculated results, Fe showed a strong positive deviation from ideal behavior across the entire composition range, while Nd showed a positive deviation in the $X_{Fe}$ mole fraction range of 0.6–1.

**Figure 10:**
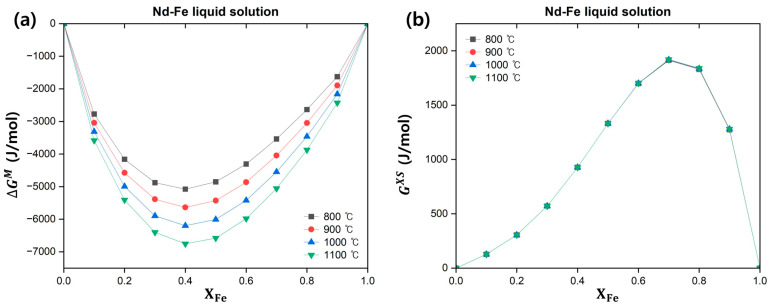

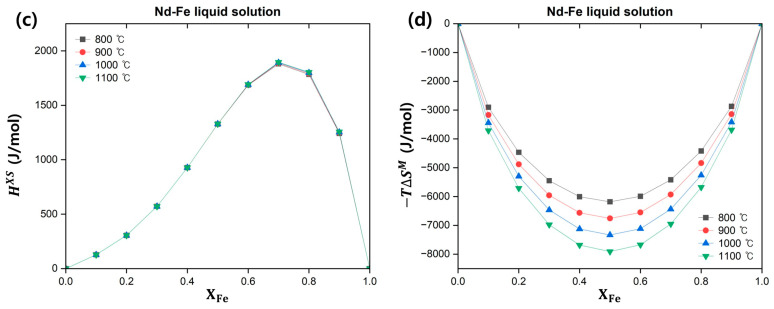
Thermodynamic parameters of the Nd–Fe liquid solution as a function of temperature and composition: (**a**) Relative integral molar Gibbs free energy of mixing; (**b**) Excess Gibbs free energy; (**c**) Excess enthalpy; (**d**) Entropy of mixing-temperature term.

The thermodynamic functions of the Nd–Fe liquid solution were plotted as a function of temperature and composition (Figure 10). Figure 10a shows the relative integral molar Gibbs free energy of mixing for the Nd–Fe liquid solution as a function of temperature and composition. The two components, Nd and Fe, spontaneously formed a solution across the entire composition range at temperatures between 800 and 1100 °C, with the most stable state occurring at $X_{Fe}$ = 0.4. Figure 10b,c show the excess Gibbs free energy and enthalpy, respectively, of the Nd–Fe liquid solution. In the temperature range of 800–1100 °C and across the entire composition, the Nd–Fe liquid solution was less stable than an ideal solution, and both thermodynamic functions exhibited low temperature dependence. The relative integral molar entropy of mixing of the Nd–Fe liquid solution in this temperature and composition range satisfied Equation (4). The calculated entropy of the mixing-temperature term expressed as a function of $X_{Fe}$ is shown in Figure 10d. Therefore, the Gibbs free energy of the Nd system decreased depending on the mixing entropy of the ideal solution and temperature term.

(4) $${∆S}^{M}<x_{A}{\Delta \bar{S}}_{A}^{M,id}+x_{B}{\Delta \bar{S}}_{B}^{M,id}=-R(x_{A}{lnx}_{A}+x_{B}{lnx}_{B})$$

where $x_{i}$: mole fraction of component i; and R: gas constant (J/mol·K).

**Figure 11:**
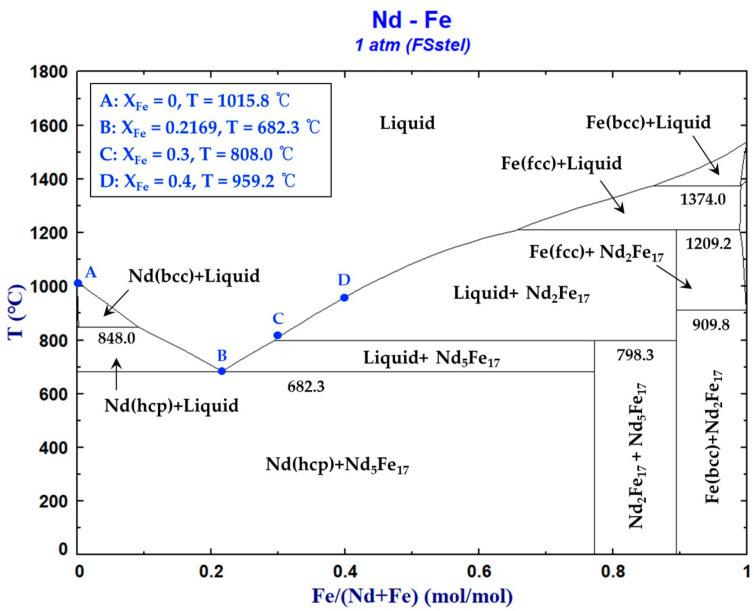
Phase diagram of the Nd–Fe binary system.

The Nd–Fe binary phase diagram investigated using the FSstel database in FactSage 8.3 is shown in Figure 11 and agrees with the diagram presented in the literature [31]. In Figure 11, points A and B represent the melting point of pure Nd (1015.85 °C) and the eutectic point ($X_{Fe}$ = 0.2169, 682.32 °C), respectively. The temperatures corresponding to points C ($X_{Fe}$ = 0.3) and D ($X_{Fe}$ = 0.4) on the liquidus lines were 808.0 and 959.2 °C, respectively. Upon equilibrium cooling, the Nd–Fe liquid solutions corresponding to compositions B, C, and D precipitated intermetallic compounds (Nd_2_Fe_17_ and Nd_5_Fe_17_) within the Nd matrix without forming solid solutions.

The compositions of Fe in the Nd–Fe liquid solution selected in this experiment were $X_{Fe}$ = 0.2169 (eutectic composition in Figure 11), 0.3, and 0.4, and the thermodynamic functions of the system at different temperatures are shown in Table 8.

#### 3.1.4. Solubility Limit of the W Crucible

**Figure 12:**
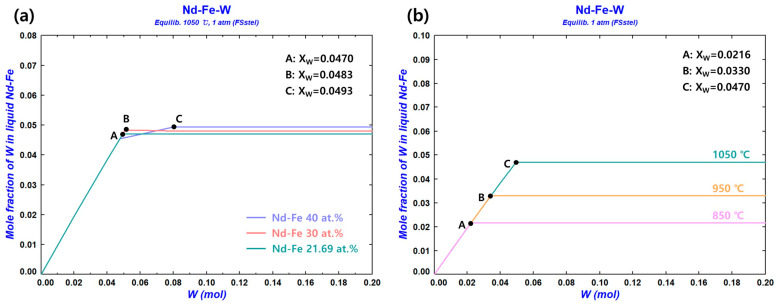
(**a**) Solubility of W in the Nd–Fe liquid solution with different compositions at 1050 °C and (**b**) at eutectic compositions for different system temperatures ($X_{W}$: mole fraction of W in the Nd–Fe liquid solution).

Thermodynamic analysis was performed using the FSstel database in FactSage 8.3 to confirm the solubility of W in the Nd–Fe liquid solution. Figure 12a shows the solubility of W in the Nd–Fe liquid solution at 1050 °C for different compositions ($X_{W}$: mole fraction of W). For the eutectic composition (Nd–Fe 21.69 at.%), the solubility limit of W was $X_{W}$ = 0.0470 (6.7579 wt.%), and W(BCC) precipitated in the saturation state. For 30 at.% Nd–Fe, the solubility limit of W was $X_{W}$ = 0.0483 (7.3396 wt.%), and W(BCC) also precipitated in the saturation state. For 40 at.% Nd–Fe, intermetallic compounds (Fe_11_W_2_ and Fe_6_W_6_) formed in the solution with the dissolution of W. The solubility limit of W was $X_{W}$ = 0.0493 (7.9199 wt.%), and W(BCC) precipitated in the saturation state. Figure 12b shows the solubility of W in the Nd–Fe liquid solution with a eutectic composition at different system temperatures. The solubility limit of W in the Nd–Fe liquid solution was $X_{W}$ = 0.0330 (4.7696 wt.%) at 950 °C and $X_{W}$ = 0.0216 (3.1421 wt.%) at 850 °C, with W(BCC) precipitating in the saturation state in both cases.

### 3.2. Metallothermic Reduction Test

#### 3.2.1. Recovery Rate of Nd

Table 9 shows the concentrations of the impurities in Nd and Fe used as the molten metal bath and in the reductant Ca. According to the analysis, C was detected in high concentrations in Nd, while the O content was relatively high in both Fe and Ca.

Table 10 shows the concentrations of the impurities in Nd and the Nd–Fe alloys recovered following metal–salt separation. Based on the N1 sample produced using only NdCl_3_ and Ca, the concentrations of C and O in Nd increased with the formation of the molten metal bath (N2). Using a mixed salt of KCl and NaCl, no significant change was observed in the concentrations of impurities except for C (N3). As the amount of Ca used in the Nd production process increased, the concentrations of Ca, K, Na, and O in Nd also increased (N3–6). With increasing Fe content in the Nd–Fe liquid solution, the concentrations of W and O in the Nd–Fe alloy increased (NF1–3). At 850–1050 °C, the concentration of W in the Nd–Fe alloy decreased as the process temperature decreased (NF1, NF4, and NF5). Unlike the solubility limits of W in the Nd–Fe liquid solution (at the wt.% level) investigated in Figure 12, the concentration of W in the Nd–Fe alloy was at the ppm level.

Table 11 shows the recovery rates of Nd when producing Nd and Nd–Fe alloys, where mass (A) represents the mass of Nd and the Nd–Fe alloys recovered following metal–salt separation, while mass (B) denotes the pure mass of Nd and the Nd–Fe alloys obtained by subtracting the impurity concentrations in Table 10 from mass (A). Mass (C) refers to the mass of Nd used in the molten metal bath, while mass (D) indicates the pure mass of Nd obtained by subtracting the impurity concentrations in Table 9 from mass (C). Similarly, mass (E) corresponds to the mass of Fe used in the molten metal bath and as a reactant, while mass (F) represents the amount of pure Fe added, which was adjusted for the impurity concentrations listed in Table 9. Consequently, the experimental mass of pure Nd produced from 100 g of NdCl_3_ satisfied the relationship: (B) − (D + F). The theoretical mass of Nd that can be obtained from 100 g of NdCl_3_ is 57.5584 g, and the recovery rate of Nd was the ratio of experimental mass-to-theoretical mass.

For sample N2, where the molten metal bath was formed, the Nd recovery rate did not significantly differ from that of the N1 sample. However, a decrease in the μm-scale metal particles present in CaCl_2_ during the metal–salt separation process was observed. This phenomenon was attributed to the absorption of reduced Nd particles into the molten metal bath during the metallothermic reduction process. Based on the N2 sample, the Nd recovery rate increased by approximately 6.5% in sample N3, which formed with the CaCl_2_–KCl–NaCl molten salt.

When a mixed salt of KCl–NaCl was used, the primary factors that could reduce the Nd recovery rate included the dissolution of NdCl_3_ into the molten salt during the reaction, which decreased the activity of NdCl_3_, and the reaction of Ca with NaCl. However, the Nd recovery rate increased, indicating that the enhanced reaction driving force due to the decreased activity of CaCl_2_ had a greater impact, thereby promoting the forward reaction over the two hindering factors. The highest Nd recovery rate (97%) was observed in sample N5, where Ca with a 1.2× stoichiometric ratio (28.7870 g) was used. However, in sample N6 with a 1.3× stoichiometric ratio (31.1859 g), the Nd recovery rate decreased compared to sample N5. Excess Ca probably reacted with the KCl–NaCl molten salt during thermal reduction, thereby leading to the dissolution of impurities (Ca, K, Na, and O) into Nd and altering the composition of the KCl–NaCl molten salt, which could subsequently hinder the dissolution of the CaCl_2_ product.

Comparing the N2 and NF1 samples, in which a molten metal bath was formed and stoichiometric Ca was used, the Nd recovery rate increased by approximately 15.0% in the NF1 sample. These results can be thermodynamically interpreted, where Nd and Fe in the Nd–Fe liquid solution exhibited positive deviations within the investigated temperature range; however, the forward reaction was promoted due to the reduced Gibbs free energy of the system. As shown in Figure 10, the Gibbs free energy of the Nd–Fe liquid solution reached its minimum at $X_{Fe}$ = 0.4. However, in the experimentally manufactured Nd–Fe alloys, the Nd recovery rate decreased as the Fe composition in the Nd–Fe liquid solution increased (NF1–3).

For the Nd–Fe alloys that were manufactured at 850–1050 °C with a target composition of 21.69 at.% Fe (NF1, NF4, and NF5), the Nd reduction rate was proportional to the temperature. These phenomena were probably caused by increases in the melting point and viscosity of the solution due to increasing Fe content or decreasing process temperatures in the Nd–Fe liquid solution, thereby restricting the transport of materials and preventing thermodynamic equilibrium. Thus, the Fe content for reducing the process temperature in the Nd manufacturing process was suitable in terms of eutectic composition when considering the Nd reduction rate. However, further research is necessary to derive the optimal process temperature considering both energy efficiency and Nd reduction rate.

#### 3.2.2. Microstructure of the Nd–Fe Alloys

**Figure 13:**
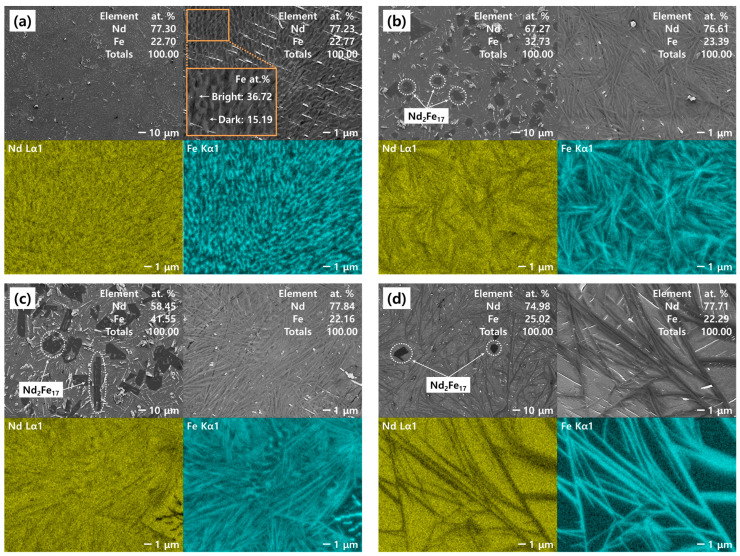
SEM images of the air-cooled Nd–Fe alloys following the metallothermic reduction process (target composition, processing temperature): (**a**) Nd–Fe 21.69 at.%, 1050 °C; (**b**) Nd–Fe 30 at.%, 1050 °C; (**c**) Nd–Fe 40 at.%, 1050 °C; (**d**) Nd–Fe 21.69 at.%, 850 °C.

Figure 13a shows the SEM image of the Nd–Fe alloy produced at 1050 °C with a target composition of 21.69 at.% Fe. At a magnification of 500×, no intermetallic compounds were observed within the Nd–Fe alloy. At 5000× magnification, the matrix of the alloy was distinguished into bright and dark regions at 36.72 and 15.19 at.% Fe, respectively. Figure 13b,c show the SEM images of the Nd–Fe alloys produced at 1050 °C, with target compositions of 30 and 40 at.% Fe, respectively. In both alloys, dark and irregularly shaped intermetallic compounds (Nd_2_Fe_17_) were observed within the matrix at 500× magnification. Notably, the contents of these intermetallic compounds increased with increasing Fe content. Moreover, the matrix composition of the Nd–Fe alloy was similar to the eutectic composition, regardless of the Fe content (21.69, 30, and 40 at.%) in the Nd–Fe liquid solution. This phenomenon can be interpreted as a result of natural air cooling. The supersaturation in the Nd–Fe liquid solution caused by non-equilibrium cooling probably led to the precipitation of intermetallic compounds and a sharp decrease in the diffusion rate, solidifying the remaining liquid phase near the eutectic composition.

Figure 13d shows the SEM image of the Nd–Fe alloy prepared at 850 °C with a target composition of 21.69 at.% Fe. The Nd_2_Fe_17_ compound, which was not observed in the alloy prepared at 1050 °C under eutectic composition conditions (Figure 13a), was detected at 500× magnification. This result was likely due to a decrease in reaction driving force and an increase in solution viscosity caused by the reduction in processing temperature. These two factors led to a decreased Nd reduction rate during the reaction, resulting in an increased Fe composition in the Nd–Fe liquid solution and consequently promoting the precipitation of intermetallic compounds during cooling. Compared to the SEM image shown in Figure 13a, the matrix of the alloy at 5000× magnification exhibited a relatively uneven distribution of Fe. This was likely due to the rapid approach to the solidification point during cooling, which limited the uniform diffusion of Fe within the microstructure.

#### 3.2.3. Salt Analysis

**Figure 14:**
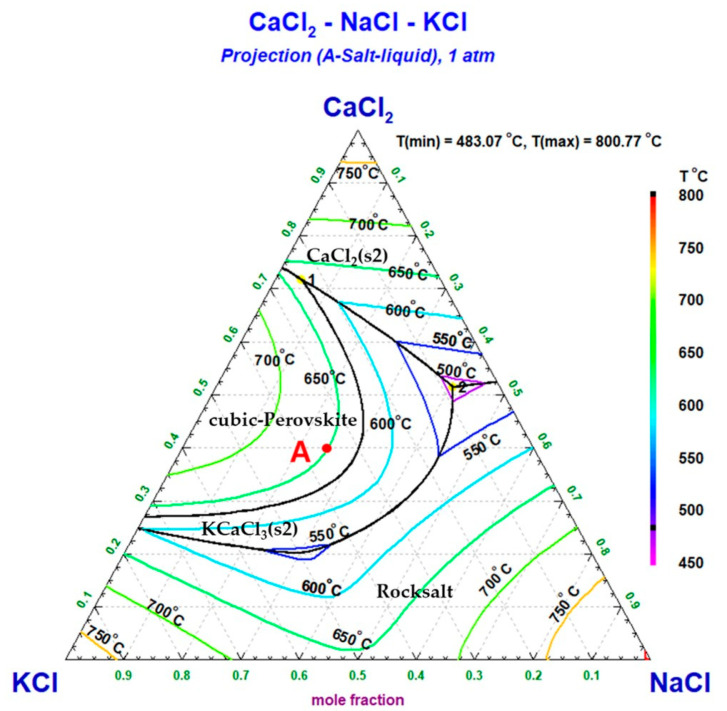
Liquidus surface projection of the ternary CaCl_2_–KCl–NaCl system. Point A: composition $X_{{CaCl}_{2}}=0.4\;(X_{KCl}:X_{NaCl}=6:4)$.

The CaCl_2_–KCl–NaCl ternary phase diagram presented in Figure 14 was calculated using the FTsalt database in FactSage 8.3. Point A represents the equilibrium composition $X_{{CaCl}_{2}}=0.4\;(X_{KCl}:X_{NaCl}=6:4)$ of the ternary molten salt, with a melting point of approximately 650 °C. Within the temperature range of 800–1100 °C, the product CaCl_2_ dissolved in the KCl–NaCl molten salt, forming a single-phase liquid solution. Compared to the melting point of pure CaCl_2_ (771.8 °C), the decreased melting point of the molten salt due to the formation of the ternary system reduced the process temperature in the Nd–Fe alloy manufacturing process.

**Figure 15:**
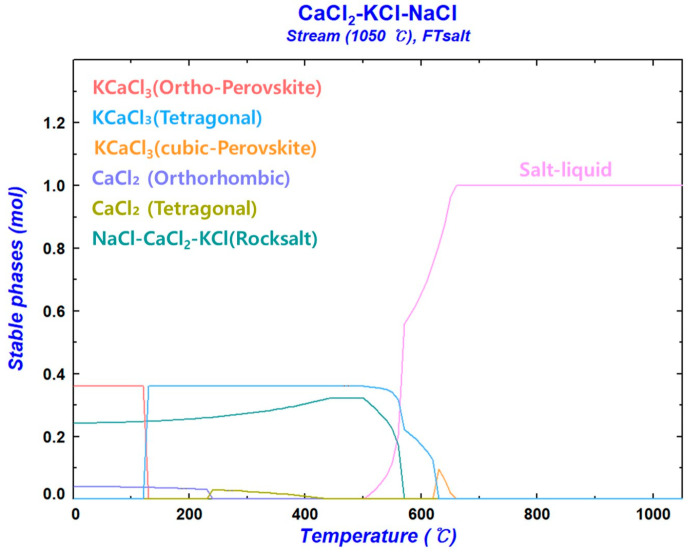
Molar quantities of the stable phases within the system as a function of temperature during equilibrium cooling from 1050 °C to room temperature for 1 mol of CaCl_2_–KCl–NaCl molten salt at $X_{{CaCl}_{2}}=0.4\;(X_{KCl}:X_{NaCl}=6:4)$.

Figure 15 illustrates the molar quantities of the stable phases within the system as a function of temperature during equilibrium cooling from 1050 °C to room temperature for 1 mol of CaCl_2_–KCl–NaCl molten salt at $X_{{CaCl}_{2}}=0.4\;(X_{KCl}:X_{NaCl}=6:4)$. The thermodynamic analysis was conducted using the stream module of the condensed system in FactSage 8.3. The stable phases constituting the chloride system at room temperature were KCaCl_3_, CaCl_2_, and rock salt (NaCl > 99.2%).

**Figure 16:**
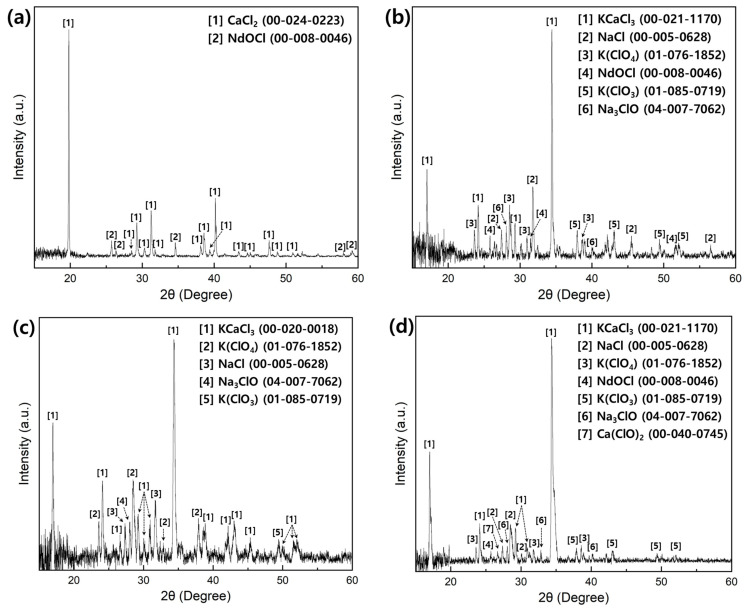
XRD patterns of CaCl_2_ and CaCl_2_–KCl–NaCl generated during the Nd and Nd–Fe alloy manufacturing process: (**a**) condition N1; (**b**) condition N3; (**c**) condition N5; (**d**) condition N6 in Table 3.

Figure 16 shows the XRD patterns of CaCl_2_ and CaCl_2_–KCl–NaCl generated during the Nd and Nd–Fe alloy manufacturing process. Figure 16a shows the XRD pattern of the chloride produced under condition N1 listed in Table 3. The major phase was CaCl_2_, with a minor peak corresponding to NdOCl. This result suggested that NdCl_3_, which was not recovered as metal, may have reacted with oxygen during the reduction process and dissolved into the molten CaCl_2_. Thus, the high concentration of oxygen in the molten salt could function as a factor reducing the recovery rate of Nd during the reaction. Figure 16b shows the XRD pattern of the chloride produced under condition N3 listed in Table 3. The major phases detected were KCaCl_3_ and NaCl, and oxygen in the chloride was present as NdOCl, K(ClO_4_), K(ClO_3_), and Na_3_ClO. Figure 16c shows the XRD pattern of the chloride produced under condition N5 presented in Table 3. The major phases were similar to those under condition N3; however, the intensity of KCaCl_3_ increased relative to N3, and no NdOCl phase was detected. The presence or absence of the NdOCl phase could be related to the Nd recovery rate. The highest Nd recovery rate was observed under condition N5, indicating that the Nd chloride content dissolved in the molten salt decreased. Thus, reducing both the oxygen concentration in the system and the composition of NdOCl in the molten salt could increase the Nd recovery rate. Figure 16d shows the XRD pattern of the chloride produced under condition N6 listed in Table 3. Although we anticipated that excessive Ca usage would suppress the formation of NdOCl, NdOCl and Ca(ClO)_2_ phases were detected. This result can be interpreted in relation to the decreased Nd recovery rate compared to the N5 condition, as explained in Table 11. The use of Ca above a certain level likely changed the composition of the CaCl_2_–KCl–NaCl molten salt, inhibiting the forward reaction and consequently leading to the formation of NdOCl.

### 3.3. GHG Emissions

#### 3.3.1. Production of NH_4_Cl and Ca

The stoichiometric amounts of NdCl_3_ and Ca used for Nd production were calculated using the reactions presented in Table 5.

The amount of NH_4_Cl required for NdCl_3_ production was calculated based on the equilibrium reaction formula between _2_O_3_ and NH_4_Cl shown in Equation (5). The chlorination reaction proceeds with the decomposition of the intermediate product NdOCl at a temperature of 306.46 °C, and NdCl_3_, NH_3_ gas, and H_2_O gas are generated [27].

(5) $${Nd}_{2}O_{3(s)}+{2NH}_{4}{Cl}_{\left(s\right)}={2NdOCl}_{\left(s\right)}+{2NH}_{3(g)}+H_{2}O_{\left(g\right)}\;{NdOCl}_{\left(s\right)}+{2NH}_{4}{Cl}_{\left(s\right)}={NdCl}_{3(s)}+{2NH}_{3(g)}+H_{2}O_{\left(g\right)},\;T\;=306.47\;{}^{\circ}C$$

The amount of NH_3_ gas required for NH_4_Cl production was derived from the reaction formula between NH_3_ and HCl gases as shown in Equation (6) [28]. In addition, the amount of H_2_ gas used for NH_3_ synthesis was derived through the Haber-Bosch process shown in Equation (7) [29]. The amount of CH_4_ used for H_2_ gas production was calculated based on the steam methane reforming and water-gas shift processes presented in Equation (8).

(6) $${NH}_{3(g)}+{HCl}_{\left(g\right)}={NH}_{4}{Cl}_{\left(s\right)}$$

(7) $$N_{2(g)}+{3H}_{2\left(g\right)}={2NH}_{3(g)}$$

(8) $${CH}_{4(g)}+H_{2}O_{\left(g\right)}={CO}_{\left(g\right)}+{3H}_{2(g)}\;{CO}_{\left(g\right)}+H_{2}O_{\left(g\right)}={CO}_{2(g)}+H_{2(g)}$$

Ca production can be divided into two stages. The first stage decomposes calcium carbonate (CaCO_3_) at a high temperature to generate CaO and CO_2_ gas, as shown in Equation (9) [32]. In the second stage, the produced CaO reacts with Al under negative pressure to obtain Ca vapor, as shown in Equation (10) [33]. The reaction was expressed based on the conditions that yielded the highest Ca recovery rate in Ref. [33].

(9) $${CaCO}_{3(s)}={CaO}_{\left(s\right)}+{CO}_{2\left(g\right)}$$

(10) $${4CaO}_{\left(s\right)}+{2Al}_{\left(l\right)}={CaOAl}_{2}O_{3(s)}+{3Ca}_{\left(g\right)}$$

As shown in Table 5, the mass of NdCl_3_ and Ca required to produce 1 kg of Nd were 1.737 and 0.417 kg, respectively. The amount of CO_2_ emissions generated during the production process of these two raw materials accord-ing to Equations (5)–(10) is shown in Table 12. In this study, the CO_2_ emissions generated during the production of NH_4_Cl and Ca were 0.343 and 0.610 kg, respectively, resulting in a total CO_2_ emission of 0.953 kg.

Therefore, the CO_2_e value of this process for producing 1 kg of Nd was 0.953 kg, which is approximately 32.1% lower than the CO_2_e (1.403 kg) of the existing electrorefining process shown in Table 2.

#### 3.3.2. Recycling of NH_3_ Gas

The NH_3_ gas generated in the chlorination process undergoes a physical dissolution and acid-base neutralization reaction with HCl in an aqueous solution as shown in Equation (11). In this process, ammonium ions (${NH}_{4}^{+}$) and chloride ions (${Cl}^{-}$) are generated [28].

(11) $${NH}_{3(g)}={NH}_{3\left(aq\right)},\;{∆G}_{298K}=-2.4\;kJ\;{NH}_{3(aq)}+{HCl}_{\left(aq\right)}={NH}_{4(aq)}^{+}+{Cl}_{\left(aq\right)}^{-},\;{∆G}_{298K}=-13.6\;kJ$$

**Figure 17:**
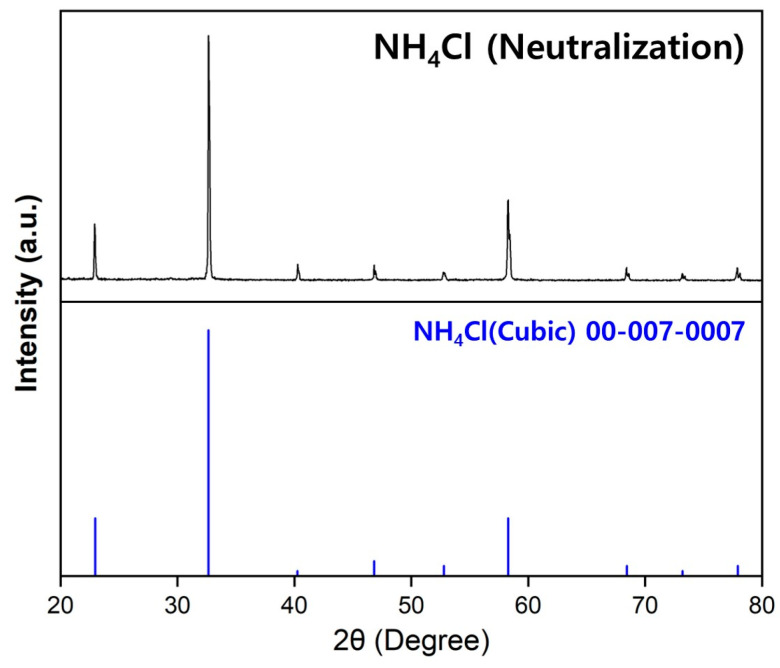
XRD pattern of NH_4_Cl synthesized by the reaction of NH_3_, generated during the chlorination process of Nd_2_O_3_ with NH_4_Cl, with HCl.

Figure 17 shows the XRD pattern of the product, which was obtained using HCl in the chlorination process, following drying at 150 °C for 24 h. The substance was identified as single-phase NH_4_Cl, and the NH_3_ gas that re-synthesized into NH_4_Cl was 72.6% of the theoretical value. These results suggest the possibility of reducing the amount of CO_2_ emissions generated during the production of NH_4_Cl (Table 12), and indicate that a higher re-synthesis rate can be achieved using equipment specialized in gas collection

## 4. Conclusions

This study aimed to identify the thermodynamic mechanism of the metallother-mic reduction reaction between anhydrous NdCl_3_ and Ca and to derive factors that could improve Nd recovery and reduce the process temperature..

The optimal composition of the CaCl_2_–KCl–NaCl molten salt that minimizes the Gibbs free energy of CaCl_2_ was $X_{{CaCl}_{2}}=0.4\;(X_{KCl}:X_{NaCl}=6:4)$, and the relative integral molar Gibbs free energy of mixing was ${\Delta G}_{800{}^{\circ}C}^{M}=-14.5$ kJ/mol and ${\Delta G}_{1100{}^{\circ}C}^{M}=-16.6$ kJ/mol at 800–1100 °C. At 1050 °C, the formation of this ternary molten salt resulted in a 6.5% increase in Nd recovery compared to the stoichiometric reaction between NdCl_3_ and Ca. Under the conditions of molten metal bath and CaCl_2_–KCl–NaCl molten salt formation at 1050 °C, the highest Nd recovery yield (97.0%) was obtained when 1.2× (by mass) the stoichiometric amount of Ca was used. However, the purity of Nd was 98.7%, caused by contamination from the molten metal bath. When the concentration of Ca used was increased to 1.3×, the concentrations of impurities (Ca, K, Na, and O) in Nd increased, and its recovery rate decreased compared to when 1.2× the amount of Ca was used. After failing to be converted into metal, NdCl_3_ reacted with oxygen in the system to form NdOCl, and it dissolved into the molten salt system during the reaction process.

At a temperature of 1050 °C, the Nd–Fe alloy (purity: 99.6%) produced with the aim of achieving a eutectic composition exhibited an Nd recovery rate of 96.3%, which represented a 15.0% increase compared to the stoichiometric reaction between NdCl_3_ and Ca. The mole fraction of Fe ($X_{Fe}$) that minimized the Gibbs free energy of the Nd–Fe liquid solution at 800–1100 °C was 0.4. However, an increase in the Fe content in the actual solution decreased the Nd recovery rate. At a temperature of 850 °C, the Nd–Fe alloy (purity: 99.6%) produced with the aim of achieving a eutectic composition exhibited an Nd recovery rate of 89.8%.

The CO_2_ emissions generated during the production of NH_4_Cl and Ca, which were used as raw materials, was 0.953 kg per 1 kg of Nd produced. This value represents a 32.1% reduction compared to that of the conventional Nd electrowinning process (CO_2_e = 1.403 kg). The capture rate of NH_3_ gas generated during the chlorination process was 72.6%, suggesting that the CO_2_ emissions from this process could be further reduced in the future.

## Figures and Tables

**Table 1:** GWP_100_ of CF_4_ and C_2_F_6_ [10].

GHG	Chemical Formula	Lifetime (Years)	GWP_100_
Carbon dioxide	CO_2_	-	1
Carbon tetrafluoride (PFC–14)	CF_4_	50,000	7390
Hexafluoroethane (PFC–116)	C_2_F_6_	10,000	12,200

**Table 2:** GHG emissions from the production of 1 kg of Nd during the electrowinning process.

GHG	Emissions	GWP_100_	CO_2e_ (kg)	
			Individual	Totals
CO	0.211 kg	-	-	1.403
CO_2_	0.055 kg	1	0.387	
CF_4_	0.118 g	7390	0.872	
C_2_F_6_	0.0118 g	12,200	0.14396	

**Table 3:** Experimental conditions.

No.	Temp. (°C)	Time (h)	Reactants (g)					Molten Metal Bath			
								Mass (g)		Mole Fraction	
			NdCl_3_	Ca	KCl	NaCl	Fe	Nd	Fe	$X_{Nd}$	$X_{Fe}$
N1	1050	4	100	23.9891	-	-	-	-	-	-	-
N2	1050	4	100	23.9891	-	-	-	100.6151	-	1	0
N3	1050	4	100	23.9891	40.1612	20.9889		100.0425	-	1	0
N4	1050	4	100	26.3880	40.1612	20.9889		100.7577	-	1	0
N5	1050	4	100	28.7870	40.1612	20.9889		100.2423	-	1	0
N6	1050	4	100	31.1859	40.1612	20.9889		100.8409	-	1	0
NF1	1050	4	100	23.9891	-	-	6.1723	90.6053	9.7160	0.7831	0.2169
NF2	1050	4	100	23.9891	-	-	9.5505	85.9442	14.2604	0.7	0.3
NF3	1050	4	100	23.9891	-	-	14.8563	79.8113	20.5999	0.6	0.4
NF4	950	4	100	23.9891	-	-	6.1723	90.6322	9.7189	0.7831	0.2169
NF5	850	4	100	23.9891	-	-	6.1723	90.7136	9.7276	0.7831	0.2169

**Table 4:** Equilibrium compositions ($X_{i}$: mole fraction of component *i*).

**No.**	Nd/Nd–Fe Liquid Solution				Molten Salt					
	Nd (g)	NdFe (g)	$X_{Nd}$	$X_{Fe}$	CaCl_2_ (g)	KCl (g)	NaCl (g)	$X_{{CaCl}_{2}}$	$X_{KCl}$	$X_{NaCl}$
N1	57.5584	-	1	0	66.4307	-	-	1	0	0
N2	158.1735	-	1	0	66.4307	-	-	1	0	0
N3	157.6009	-	1	0	66.4307	40.1612	20.9889	0.40	0.36	0.24
N4	158.3161	-	1	0	66.4307	40.1612	20.9889	0.40	0.36	0.24
N5	157.8007	-	1	0	66.4307	40.1612	20.9889	0.40	0.36	0.24
N6	158.3993	-	1	0	66.4307	40.1612	20.9889	0.40	0.36	0.24
NF1	-	164.0520	0.7831	0.2169	66.4307	-	-	1	0	0
NF2	-	167.3135	0.7	0.3	66.4307	-	-	1	0	0
NF3	-	172.8259	0.6	0.4	66.4307	-	-	1	0	0
NF4	-	164.0818	0.7831	0.2169	66.4307	-	-	1	0	0
NF5	-	164.1719	0.7831	0.2169	66.4307	-	-	1	0	0

**Table 5:** Standard Gibbs free energy change for the reaction of NdCl_3_ with Ca calculated using the FactPS database in the FactSage 8.3 software.

Reaction Formula	$∆G{}^{\circ}\left(T\right)$ (J)	Temp. Range (K)
2NdCl_3(l)_ + 3Ca_(s)_→2Nd_(s)_ + 3CaCl_2(s)_	1,279,620 − 11,773.1 × T − 1.2 × 10^−1^ × T^2^ − 1.9 × 10^7^ × T^−1^ −3.6 × 10^−6^ × T^3^ − 5.9 × 10^5^ × lnT + 1.8 × 10^5^ × T^0.5^ + 1232.3 × TlnT	1032–1045 (CaCl_2_: Rutile→Liquid)
2NdCl_3(l)_ + 3Ca_(s)_→2Nd_(s)_ + 3CaCl_2(l)_	−81,266 + 684.2 × T + 4.8 × 10^−2^×T^2^ − 6.7 × 10^6^ × T^−1^ − 3.6 × 10^−6^ × T^3^ −5.1 × 10^4^ × lnT − 91.7 × TlnT	1045–1115 (Ca: β→Liquid)
2NdCl_3(l)_ + 3Ca_(l)_→2Nd_(s)_ + 3CaCl_2(l)_	−341,380 − 300.5 × T − 8.4 × 10^−3^ × T^2^ − 3.6 × 10^−6^ × T^3^ + 44.8 × TlnT	1115–1128 (Nd: HCP→BCC)
2NdCl_3(l)_ + 3Ca_(l)_→2Nd_(s)_ + 3CaCl_2(l)_	−363,472 + 11.5 × T + 1.2 × TlnT	1128–1289 (Nd: BCC→Liquid)
2NdCl_3(l)_ + 3Ca_(l)_→2Nd_(l)_ + 3CaCl_2(l)_	−360,082 + 69.4 × T − 7.3 × TlnT	1289–1774 (Ca: Liquid→Gas)

**Table 6:** Thermodynamic parameters of the CaCl_2_–KCl–NaCl liquid solution with a composition of $X_{{CaCl}_{2}}=0.4\;(X_{KCl}:X_{NaCl}=6:4)$ at different system temperatures.

**Temp. (°C)**	$X_{{CaCl}_{2}}$	$X_{KCl}$	$X_{NaCl}$	$\gamma_{{CaCl}_{2}}$	$\gamma_{KCl}$	$\gamma_{NaCl}$	${∆G}^{M}$ (J/mol)	$G^{XS}$ (J/mol)
800	0.40	0.36	0.24	0.4990	0.5034	0.9025	−14,514	−4905
900				0.5393	0.5536	0.9098	−15,211	−4707
1000				0.5756	0.5991	0.9159	−15,913	−4514
1100				0.6086	0.6403	0.9210	−16,619	−4325

**Table 7:** Activity of Nd and Fe in the Nd–Fe liquid solution at different temperatures calculated using the FSstel database in the FactSage 8.3 software.

$X_{Fe}$	800 °C		900 °C		1000 °C		1100 °C	
	$a_{Fe}$	$a_{Nd}$	$a_{Fe}$	$a_{Nd}$	$a_{Fe}$	$a_{Nd}$	$a_{Fe}$	$a_{Nd}$
0	0	1	0	1	0	1	0	1
0.1	0.1172	0.8985	0.1156	0.8986	0.1143	0.8987	0.1132	0.8988
0.2	0.2517	0.7883	0.2469	0.7893	0.2428	0.7901	0.2394	0.7908
0.3	0.4094	0.6714	0.3987	0.6738	0.3899	0.6758	0.3825	0.6775
0.4	0.5772	0.5588	0.5594	0.5622	0.5449	0.5651	0.5328	0.5675
0.5	0.7275	0.4632	0.7048	0.4661	0.6862	0.4686	0.6706	0.4708
0.6	0.8350	0.3922	0.8123	0.3926	0.7936	0.3930	0.7780	0.3933
0.7	0.8943	0.3461	0.8765	0.3416	0.8617	0.3379	0.8492	0.3348
0.8	0.9209	0.3175	0.9104	0.3053	0.9015	0.2953	0.8939	0.2871
0.9	0.9424	0.2755	0.9387	0.2534	0.9356	0.2361	0.9330	0.2221
1	1	0	1	0	1	0	1	0

**Table 8:** Relative integral molar Gibbs free energy of mixing and excess Gibbs free energy as a function of system temperature and the composition change of the Nd–Fe liquid solution.

$X_{Fe}$	800 °C		900 °C		1000 °C		1100 °C	
	${∆G}^{M}$ (J/mol)	$G^{XS}$ (J/mol)	${∆G}^{M}$ (J/mol)	$G^{XS}$ (J/mol)	${∆G}^{M}$ (J/mol)	$G^{XS}$ (J/mol)	${∆G}^{M}$ (J/mol)	$G^{XS}$ (J/mol)
0.2169	−4323	343	−4758	343	−5192	343	−5627	343
0.3	−4879	572	−5387	572	−5895	572	−6403	572
0.4	−5077	928	−5637	928	−6196	928	−6756	928

**Table 9:** Concentrations of the impurities in Nd and Fe used as the molten metal bath and in the reductant Ca.

Sample	Ca	K	Na	W	Cl	C	O	Total (wt.)
Nd	0.0739	0.0366	0.0344	0.0424	0.0415	0.9207	0.0452	1.1948
Fe	0.0888	0.0577	0.0038	0.0004	N.D.	0.0120	0.4074	0.5701
Ca	-	0.0124	0.0252	0.0017	N.D.	0.0212	2.3127	2.3732

**Table 10:** Concentrations of the impurities in Nd and the Nd–Fe alloys.

Sample	Ca	K	Na	W	Cl	C	O	Total (wt.%)
N1	0.1234	0.0024	0.0200	0.0165	0.0219	0.0420	0.0016	0.2278
N2	0.1229	0.0116	0.0210	0.0220	0.0406	0.1403	0.0507	0.4092
N3	0.1201	0.0135	0.0268	0.0268	0.0634	0.2007	0.0626	0.5140
N4	0.2069	0.0327	0.0209	0.0225	0.0498	0.2056	0.1010	0.6394
N5	0.6959	0.0921	0.0584	0.0283	0.0507	0.2029	0.1864	1.3148
N6	1.4410	0.5074	0.1586	0.0333	0.0669	0.2748	0.2913	2.7734
NF1	0.0985	0.0157	0.0169	0.0323	0.0506	0.1824	0.0466	0.4430
NF2	0.1186	0.0108	0.0165	0.0467	0.0471	0.1909	0.0487	0.4793
NF3	0.1517	0.0172	0.0151	0.0584	0.0543	0.1662	0.1157	0.5787
NF4	0.1026	0.0138	0.0175	0.0286	0.0407	0.2069	0.0519	0.4621
NF5	0.1234	0.0119	0.0180	0.0242	0.0335	0.1945	0.0291	0.4346

**Table 11:** Calculation of Nd recovery rate: (A) Masses of Nd and the Nd–Fe alloys measured following salt separation; (B) Masses of pure Nd and the Nd–Fe alloys excluding impurities; (C, E) Amount used in the experiments; (D, F) Amount excluding impurities.

No.	Nd/Nd–Fe Alloy (g)		Nd (Bath) (g)		Fe (Bath + Reactant) (g)		Nd Recovery	
	(A)	(B)	(C)	(D)	(E)	(F)	(g)	(%)
N1	46.1052	46.0002	-	-	-	-	46.0002	79.9191
N2	146.8214	146.2207	100.6151	99.4130	-	-	46.8077	81.3220
N3	150.1836	149.4117	100.0425	98.8472	-	-	50.5645	87.8490
N4	153.3236	152.3432	100.7577	99.5539	-	-	52.7893	91.7143
N5	156.9405	154.8771	100.2423	99.0447	-	-	55.8324	97.0013
N6	158.0924	153.7079	100.8409	99.6361	-	-	54.0718	93.9424
NF1	161.4635	160.7482	90.6053	89.5228	15.8883	15.7977	55.4277	96.2981
NF2	162.6488	161.8692	85.9442	84.9174	23.8109	23.6752	53.2767	92.5611
NF3	166.9262	165.9603	79.8113	78.8578	35.4562	35.2541	51.8484	90.0796
NF4	158.6776	157.9444	90.6322	89.5494	15.8912	15.8006	52.5944	91.3757
NF5	157.8216	157.1357	90.7136	89.6298	15.8999	15.8093	51.6967	89.8160

**Table 12:** CO_2_ emissions from the production process of 1 kg of Nd.

Components	1.737 kg NdCl_3_					0.417 kg Ca			Total CO_2_ Emissions
	NH_4_Cl	NH_3_	H_2_	CH_4_	CO_2_	CaO	CaCO_3_	CO_2_	
(kg)	1.113	0.354	0.031	0.125	0.343	0.778	1.388	0.610	0.953

## Data Availability

The original contributions presented in this study are included in the article. Further inquiries can be directed to the author.
